# A Bi-Directional Carrier Sense Collision Avoidance Neighbor Discovery Algorithm in Directional Wireless Ad Hoc Sensor Networks

**DOI:** 10.3390/s19092120

**Published:** 2019-05-07

**Authors:** Annan Yang, Bo Li, Zhongjiang Yan, Mao Yang

**Affiliations:** School of Electronics and Information, Northwestern Polytechnical University, Xi’an 710072, China; yangannan1123@mail.nwpu.edu.cn (A.Y.); libo.npu@nwpu.edu.cn (B.L.); yangmao@nwpu.edu.cn (M.Y.)

**Keywords:** directional wireless ad hoc sensor networks, neighbor discovery, bi-directional carrier sense, multi-subchannel, scan based algorithm (SBA)

## Abstract

From the perspective of media protocol control and routing of directional wireless ad hoc sensors networks, neighbor discovery protocol is an important problem to be solved first. In the past period of time, some methods have been studied on neighbor discovery protocol, but they have a common defect of link collision. The collision is caused by mutual interference of multiple transmitting nodes which are in one reception beam of the receiving node. To solve this problem, we propose a neighbor discovery algorithm using a bi-directional carrier sense collision avoidance and multi subchannels based on a scan-based algorithm (BD-SBA). Based on a scan-based algorithm (SBA), bi-directional carrier sense of the BD-SBA algorithm is performed in the first broadcast step which can reduce the collision of broadcasting the scanning request (SREQ) frames. In the second step (the reply step), the mechanism of multiple subchannels and multiple slots is applied to reduce the collision of the scanning response (SRES) frames. From the analysis and simulation, we can see that nodes using proposed algorithm can discover their neighbor nodes in fewer time. Moreover, the proposed algorithm has better performance for different beamwidths and densely distributed scenes. So it has great significance in engineering application.

## 1. Introduction

Different from traditional wireless sensor networks that require base stations, wireless ad hoc sensor networks (WASNs) can quickly and spontaneously be deployed without fixed communication infrastructures [1]. There are dozens or even hundreds of nodes in WASNs, which can be used for sensor detection, communication or route establishment. Without considering connection and security constraints, these nodes can communicate with each other, so the WASNs have great flexibility. Because of the great flexibility, WASNs have a variety of applications, such as intelligent transportation systems, battlefield surveillance and communications systems, environment monitoring systems, etc. [2].

Previous research on WASNs consider omnidirectional antennas, which not only have limited transmission distance, but also cause interference between nodes. In recent years, with the development of mobile communication and antenna technology, directional antennas have become a hot spot. In WASNs, directional antennas with beamforming not only greatly increase the frequency efficiency and system capacity, but also bring many advantages such as long communication distance and low detected probability. Therefore, the use of directional antennas is an effective way to solve the problem of wireless spatial frequency resources lack [3]. An important feature of the directional WASNs using the beamforming technique is self-configuration, which means that the deployed nodes can spontaneously form the networks. The self-configuration needs nodes to set up routing spontaneously, and neighbor discovery is the first problem to be solved [4].

### 1.1. Related Works

Neighbor discovery is the first step to discover potential single hop nodes and establish links between nodes in directional WASNs. According to the clock synchronization in the networks, the neighbor discovery algorithms can be divided into synchronous algorithms [5,6,7,8,9,10,11,12,13] and asynchronous algorithms [14,15,16]. Although the synchronous algorithms need additional hardware or GPS support, the complexity is much lower than asynchronous algorithms. At present, most of the neighbor discovery algorithms are based on synchronous algorithms, and it is so in this research.

The synchronous neighbor discovery algorithms with directional antennas are divided into two main categories: completely-random algorithms (CRA) [5,6,7,8,9] and scan-based algorithms (SBA) [10,11,12,13]. They have something in common: (1) the directions of the beams are synchronized in time; (2) the successful communication between nodes requires that straight line between them should be covered by both the transmitting beam of one node and the receiving beam of the other. (3) When multiple transmitting nodes exist in one reception beam of one receiving node, a “link collision” problem will occur. For CRA, each node selects the direction of the antenna beam completely randomly according to a certain probability. Therefore, the neighbor discovery based on CRA is memoryless and robust, and is suitable for sparse distribution and no priori scenes. However, the disadvantage of CRA is the large delay due to beam misalignment, and two neighbor nodes may not be able to discover each other in a long time. Compared to the CRA, the advantage of the SBA is that the antenna direction is scanned according to a certain rule (such as clockwise), so the beam of the two neighbor nodes can point to each other within the upper limit of time.

Due to the advantages of discover time, SBA has become the research focus of synchronous neighbor discovery algorithm. Zhang proposes a directional transmission and reception algorithm (DTRA) in [10], and the SBA algorithm is used in the neighbor discovery stage. DTRA defines a data transmitting and receiving protocol in directed ad hoc networks, which divided the whole transmission process into three stages: neighbor discovery, reservation and data transmission stage. The neighbor discovery algorithm in [10] adopts the three-way handshake mechanism, which determines the transmitting or listening mode of node according to binary form, and ensures that the nodes can discover their neighbor nodes at most $\left\lceil \log_{2}N\right\rceil$ scans (*N* is the maximal number of nodes). Based on [10], Zhang et al. compares four algorithms in [11], which are completely random algorithm with directional transmit and receive (CRA-DD), completely random algorithm with directional transmit and omnidirectional receive (CRA-DO), scan based algorithms with deterministic mode selection (SBA-D) and scan based algorithms with random mode selection (SBA-R), and the theoretical model and simulation comparison of them are given. Based on [11], the SBA algorithm was studied in [12]. The innovation of [12] is that the concept of “direction-pair” and the model of the algorithm considering interference are established, in which multiple nodes in the same reception beam of one node are considered as interference. An improved scan-based algorithm (I-SBA) algorithm is proposed in [13]. The algorithm adds a new idle mode, and can greatly reduce the interference caused by the transmitting frames in the first broadcast step of the three-way handshakes.

### 1.2. Our Contributions

Upon reviewing the existing researches, there are several unsolved problems: (1) Zhang et al. [10,11] have made a pioneering contribution to the SBA-based neighbor discovery algorithm. However, when two or more nodes are in the same reception beam of one node, the interference problem is not considered. This “link collision” problem cannot be ignored in reality, especially when the node density is large. (2) An SBA model considering interference is established in [12], but the solution is not given. (3) The method proposed by Cai et al. in [13] reduces the interference caused by the transmission of the broadcast step, but it has not solved the interference caused by the reply step.

From the existing research of neighbor discovery algorithm based on SBA, we can see that the performance bottleneck mainly exists in two aspects: Firstly, in the process of the first handshake step (the broadcast step), multiple transmitting nodes are in the same reception beam of one receiving node. The collision of transmission packets from multiple transmitting nodes will cause failure of receiving the packets. Secondly, if multiple nodes are in the receiving mode within the beam of the transmitting node, these nodes should reply with a probability of 1. Meanwhile, these transmitting packets collide and cause the failure of neighbor discovery.

With the development of antenna technology and the advancement of digital processor, bi-directional and multi-directional antennas have come out [17,18], and this type of multi-directional antennas has been applied to the design of directed network MAC protocol [19,20]. Therefore, this paper proposes a neighbor discovery algorithm using bi-directional carrier sense collision avoidance and multi subchannels based on scan based algorithm (BD-SBA). In our algorithm, before one node transmits the first handshake broadcast frame, it first listens to the carrier of the peripheral nodes within the bi-directional beam. When the channel is idle, it sets its own mode as transmitting, otherwise receiving. Because of the bi-directional carrier sense mechanism, the interference between nodes can be greatly reduced and the success probability of receiving the first handshake broadcast frame can be greatly increased. Subsequently, after receiving the first handshake frame, the node randomly select a time-frequency resource block to reply the second response frame through multi-subchannel and multi-slot. Only when two or more nodes choose the same time-frequency resource block, the collision occurred. The using of multi-subchannel and multi-slot techniques in the process of second handshake can greatly reduce the probability of the collision.

In this paper, bi-directional carrier sense collision avoidance and multi-subchannel multi-slot techniques are introduced into our BD-SBA neighbor discovery algorithm. The mathematical model and simulation result shows that our BD-SBA algorithm has excellent performance on neighbor discovery, and is insensitive to beamwidth and suitable for nodes densely distributed scene.

The remainder of this paper is organized as follows. In Section 2, we introduce the principle and basic procedure of the algorithm. In Section 3, the analytical model and simulation results of analytical model are given. The simulation of the algorithm in different scenarios is given in Section 4. Finally, we conclude this paper in Section 5.

## 2. System Model and Basic Procedure

### 2.1. System Model of Traditional SBA Algorithm

**Figure 1:**
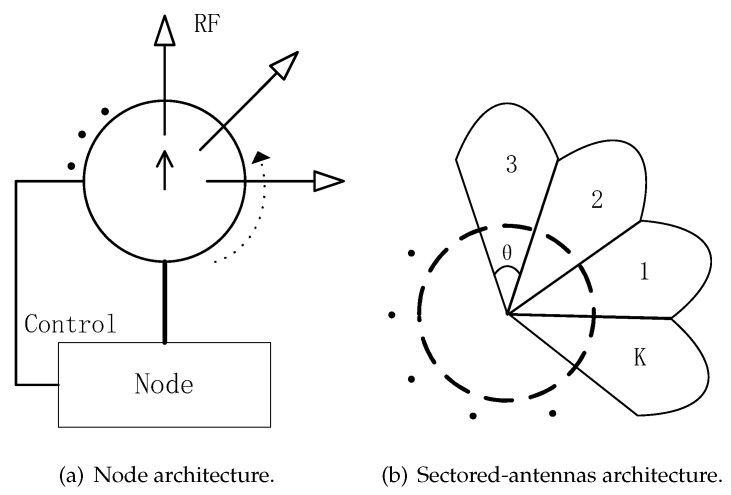
Node and sectored-antennas architecture of traditional scan-based algorithm (SBA) algorithm.

As shown in Figure 1a, a node can control some steerable directional antennas and can select one directional antenna for transmitting or receiving data. Time is slotted and nodes are synchronized by GPS or other equipments. The communication mode is half-duplex, that is, node cannot transmit and receive simultaneously. As shown in Figure 1b, for convenience of study, the beam is considered as an ideal beam in this paper, that is, there is no side lobe and tail lobe effect. We assume that the beamwidth of one sector is θ (0<θ<2π) and there are *K* sectors in one node, and $K=360{}^{\circ }/\theta$.

**Figure 2:**
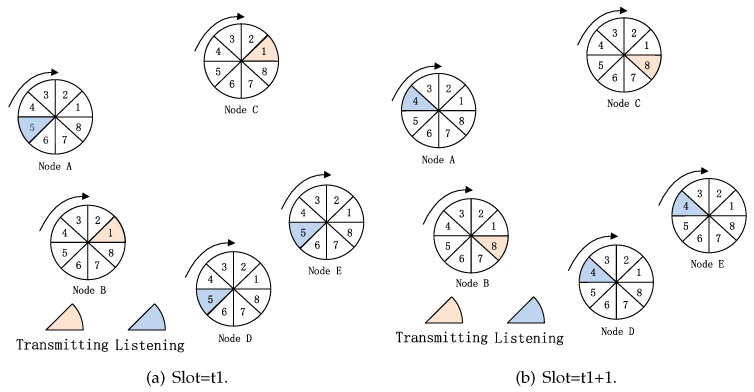
Illustration of traditional SBA algorithm.

For the traditional SBA algorithm, if the straight line connecting two nodes is contained within both of their beams and one of the two nodes is in the transmitting mode and the other is in the receiving mode, they can communicate with each other. Therefore, if the two nodes want to discover each other, they should ensure that their beam sectors must point to each other and they are in opposite transmitting/receiving mode. For the SBA algorithm, each node’s beam rotates in a pre-stored way, such as clockwise or counter-clockwise from 6 o’clock or 12 o’clock. During the procedure of beam rotation, the node can be in transmitting or receiving mode. We call the sequence of beam-pointing directions and node modes during beam rotation a scan. Figure 2 is an illustration of SBA algorithm. As shown in Figure 2a, we assume that there are five nodes A–E and their sector degree is 45°. At t1 slot, node B and node C have rotated a sector degree clockwise from 12 o’clock, and they choose the beam of sector 1. Nodes A, D and E have also rotated a sector degree clockwise from six o’clock, and they choose the beam of sector 5. The mode (transmitting or receiving) of nodes can be selected randomly with a certain probability or according to a specific rule [11]. As shown in Figure 2a, at the t1 slot, assuming that nodes B and C choose to be in the transmitting mode and node A, D and E are in the receiving mode, no nodes are discovered at the t1 slot because no beams of nodes point to each other at this time. But at t1+1 slot, the beams pointing of all the nodes in Figure 2a rotate 45° clockwise. As shown in Figure 2b, at this time, the beam pointing of node B and D point to each other and they are in opposite mode (one transmitting and one receiving). Without considering the “link collision” problem, nodes B and D can discover each other.

**Figure 3:**
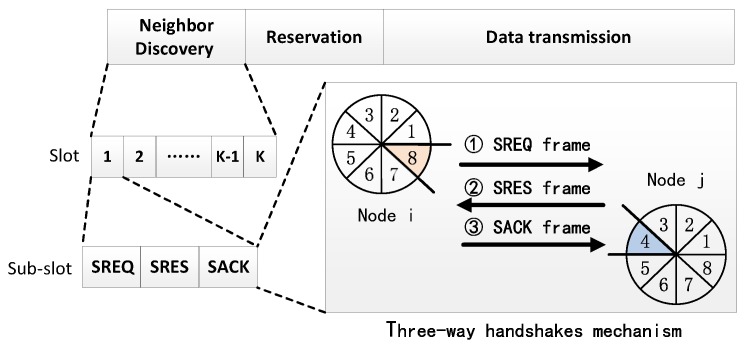
Directional transmission and reception algorithm (DTRA) method and the process of three-way handshakes mechanism.

For a pair of nodes, when their beams point to each other and their modes are opposite, they can discover each other by the way of a two-way handshaks or three-way handshakes. The choice of two or three handshakes mechanism is related to the practical application of the ad hoc network. If the nodes in the network only need to discover their neighbor nodes, two-way handshakes can be used. If more reliable communication is needed after the neighbor discovery stage, then three-way handshaks mechanism is better. A classic application of an SBA algorithm in ad hoc networks is the DTRA method which includes three stages of neighbor discovery, reservation and data transmission stage [10]. As shown in Figure 3, the DTRA method divides time into three stages: neighbor discovery, reservation and data transmission. In the neighbor discovery stage, time is divided into multiple slots, each slot represents a sector pointing at a fixed angle. As shown in Figure 3, nodes *i* and *j* have eight sectors, each sector is 45°, and the number of slots K equals eight. The beam pointing of the node rotate a sector degree each slot, and each slot can be divided into three sub-slots, which are used to transmit scanning request (SREQ) frame, scanning response (SRES) frame and scanning acknowledgement (SACK) frame. As shown in Figure 3, assuming that the node *i* and *j* is in the transmitting and receiving mode respectively. The direction of beam sector 8 of node *i* is opposite to beam sector 4 of node *j*. Firstly, node *i* transmits a SREQ frame advertising itself to potential neighbors. Node *j* listens to the transmission until it receives the SREQ frame successfully, then it will reply SRES frame to node *i*. Finally, after receiving the SRES frame correctly, node *i* replies SACK frame to node *j*. Node *i* and node *j* complete their neighbor discovery.

### 2.2. Motivation

**Figure 4:**
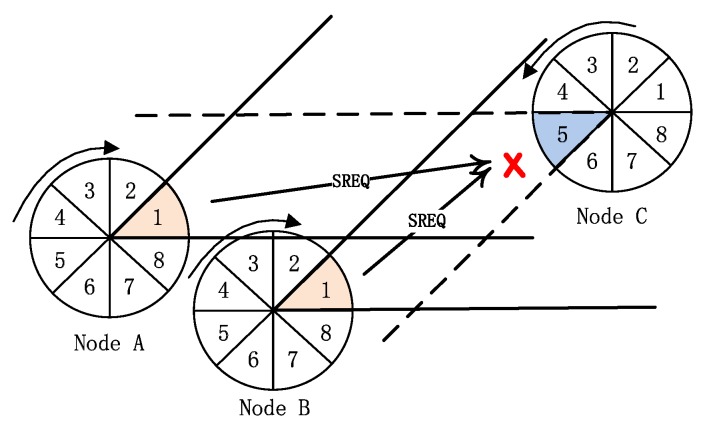
The first “link collision” problem-the collision of the first handshake.

**Figure 5:**
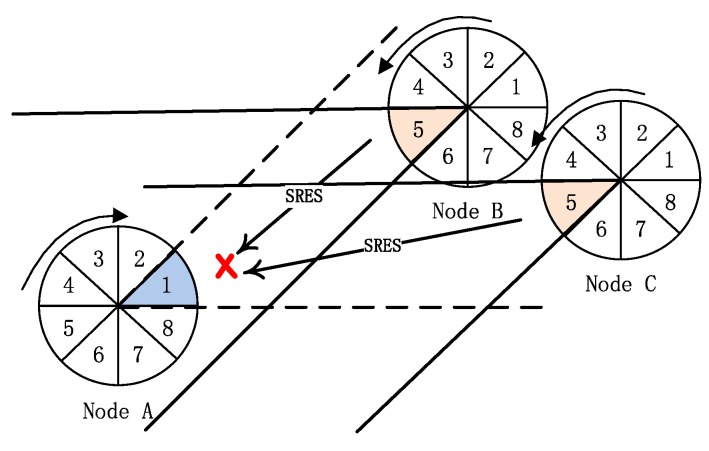
The second “link collision” problem-the collision of the second handshake.

In directional ad hoc networks, how to reduce collision in communication is the key to improve network performance. Especially, the “link collision” problem is the bottleneck that restricts the performance of neighbor discovery algorithm. For the SBA algorithm in [10,11], when nodes are distributed densely or the number of neighbor nodes is large, two kinds of “link collision” problems will have a serious impact on the algorithm performance:

1. The first “link collision” problem: during the first handshake step, there are multiple nodes in the transmitting mode in the reception beam of one node in the receiving mode. If the direction of multiple transmitting nodes’ beams is opposite to the direction of the receiving node’s beam and these nodes in transmitting mode transmit SREQ frames at the same time, the node in receiving mode will not receive any SREQ frame successfully. As shown in Figure 4, the beams of nodes A and B are in sector 1 and they transmit SREQ frames simultaneously. At this time, the beam of node C in sector 5 is opposite to the beams of nodes A and B, and it is in receiving mode. Because of the two nodes (nodes A and B) simultaneously transmitting SREQ frames in the receiving beam of node C, node C can not receive any SREQ frame. That is the first “link collision” problem in the previous SBA algorithms.
2. The second “link collision” problem: when there are multiple nodes in the beam of the node in transmitting mode and they are in the receiving mode after the first handshake, they will reply SRES frames with the probability of 1 according to the SBA algorithm. Then these second handshake frames collided and resulted in the failure of discovery. As shown in Figure 5, node A successfully transmitted the first handshake frame (SREQ frame) to node B and C, and node B and C should reply the second handshake frame (SRES frame) to node A at the same time according to the SBA algorithm. The result is that the two SRES frames transmitted to node A collided and node A can not receive any SRES frame. That is the second “link collision” problem in the previous SBA algorithms.

### 2.3. BD-SBA Algorithm Description

**Figure 6:**
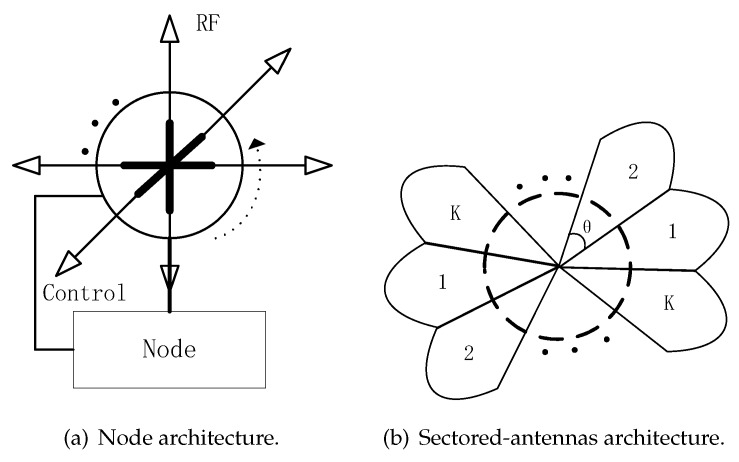
Node and sectored-antennas architecture of bi-directional (BD)-SBA algorithm.

For BD-SBA algorithm, as shown in Figure 6a, bi-directional antennas are used and the angle difference between the two antennas is 180 degrees. The node control the beam rotation of the bi-directional antennas synchronously. As shown in Figure 6b, for convenience of study, the beam is considered as an ideal beam in this paper, that is, there is no side lobe and tail lobe effect. The bi-directional antenna can transmit data in the opposite direction synchronously, while it can sense energy in the opposite direction at the same time when receiving. The beams of the nodes choose to rotate clockwise or counterclockwise, so each node only needs to rotate $180^{\circ }$ to ensure that the directions of the node and the neighbor nodes beams are opposite. Similarly, the BD-SBA algorithm in this paper still uses the three-way handshakes mechanism shown in Figure 3.

The brief process of the BD-SBA algorithm is as follows: at the beginning of scanning, each node randomly selects a value within the backoff contention window (CW) [0 CW-1] (CW is the maximum of the backoff contention window) and carry out carrier sense and backoff. The node that backoff to 0 first sets its mode to transmitting and then transmits the SREQ frame. However, the nodes that listening to the busy carrier will set their modes to receiving and prepare to receive SREQ frames. The nodes receiving SREQ frames successfully need to randomly select a time-frequency resource block on multi subchannels and multi-slot to reply SRES frames. Then the nodes receiving SRES frames successfully decide whether to transmit SACK frames according to subsequent reservation and data transmission requirement.

**Figure 7:**
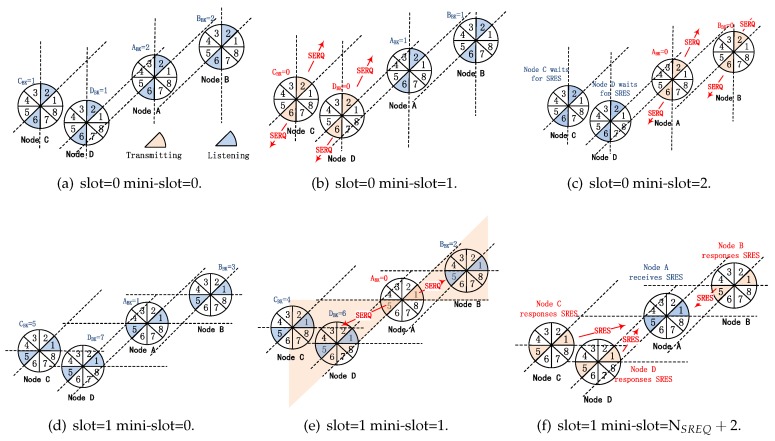
Illustration of BD-SBA algorithm.

Figure 7 is an illustration of BD-SBA algorithm. Assuming that there are only four nodes A, B, C and D in the scene, the beam sector of each node points to 12 o’clock at the beginning of scan (slot = 0) and rotates clockwise synchronously. The process is as follow:

1. As shown in Figure 7a, nodes A, B, C and D select a backoff counter value of 2, 2, 1, 1 within the range of [0,15] (assuming CW = 16) and start backoff, while performing physical carrier sense in the process of backoff.
2. As shown in Figure 7b, node C and D backoff to 0 first. Since they don’t sense any physical energy during their backoff process, they set their own modes to transmitting, and then transmit SREQ frames in sectors 2 and 6 in opposite directions simultaneously.
3. As shown in Figure 7c, subsequently, nodes A and B backoff to zero. Similarly, they transmit SREQ frames in sectors 2 and 6, respectively. At slot 0, node A, B, C and D do not receive any SRES frame because there are no nodes’ beams pointing to each other. Therefore, no neighbor nodes are discovered at slot 0.
4. As shown in Figure 7d, nodes A, B, C and D have rotated their bi-directional antennas clockwise 45 degrees when slot = 1. Their beam sectors are located in sectors 1 and 5 respectively, and the backoff counter values of 1, 3, 5, 7 are randomly selected. They start their backoff and physical carrier sensing.
5. As shown in Figure 7e, node A backoff to 0 first, setting its own mode to transmitting, and transmit SREQ frame in its bi-directional beam sector 1 and 5. Nodes B, C and D are performing physical carrier sensing and are in receiving mode. They receive SREQ frames transmitted by node A in their sector 5, 1, 1. So that node B, C and D discover node A and prepare to reply SRES frames.
6. As shown in Figure 7f, $N_{SREQ}+1$ mini-slots has passed ($N_{SREQ}$ is the number of mini-slots of SREQ frame), and nodes B, C and D have received SREQ frame, then they set their own modes to transmitting, and randomly select a time-frequency resource block to reply SRES frame in their respective sectors 5, 1 and 1. At this time, node A sets its own mode to receiving and receives SRES frames in sector 5 and 1. Then node A can discovered node B, C and D.

From Figure 7 we can see, firstly, because each node adopts the mechanism of bi-directional carrier sense collision avoidance, which greatly reduces the possibility of multiple nodes transmitting SREQ frames at the same time. For example, as shown in Figure 7e, if the traditional SBA algorithm is used and assuming node C and D are in transmitting mode and node A is in receiving mode, the SREQ frames transmitted by node C and D will collide. However, due to the mechanism of bi-directional carrier sense collision avoidance, the transmissions of SREQ frames of node B, C and D are restrained. Secondly, replying SRES frames on multi time-frequency resource blocks can reduce the impact of the second kind of “link collision” problem. For example, as shown in Figure 7f, if using a traditional SBA algorithm, node C and D reply SRES frames simultaneously, then collision will occur and node A can’t discover its neighbor node C and D.

### 2.4. Detailed Procedure of BD-SBA Algorithm

**Figure 8:**
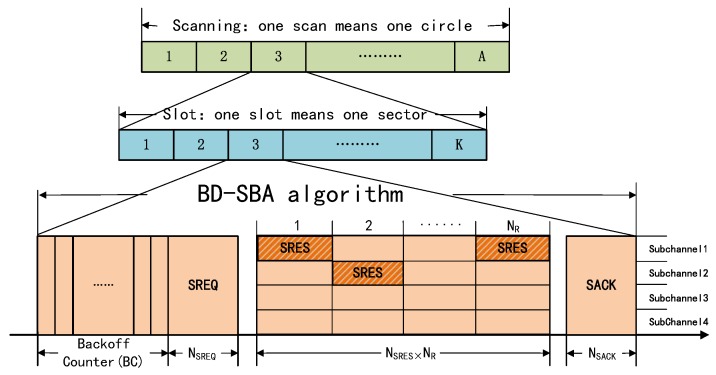
Algorithm frame structure.

As shown in Figure 8, the neighbor discovery stage is formed by A scans, which one scan means the beam of node rotates one circle. Each scan is formed by K slots and the beam points to different sectors under different slots. Each slot consists of several mini-slots. Each node maintains its own backoff counter (BC), which decreases by a mini-slot. Each frame occupies several mini-slots according to its bit information.

The detailed procedures are as follows:

1. Step 1—backoff and bi-directional carrier sense:
  Firstly, the node carries out the backoff process based on bi-directional carrier sense. The node randomly chooses a BC value in the range of [0, CW-1] and begin backoff. In the process of backoff, if the node do not listen to any physical energy transmitted by other nodes in its sector, it sets its mode to transmitting. Otherwise, it set its mode to receiving.
2. Step 2—broadcasting SREQ frame:
  All nodes in the transmitting mode broadcast SERQ frames in their sectors while all nodes in the receiving mode ready to receive these SERQ frames. The SREQ frame contains the identification and the sector index of transmitting node. SREQ frames contain $N_{SREQ}$ mini-slots.
3. Step 3—replying SRES frame:
  All the nodes that successfully receive SREQ frames must reply SRES frames with the probability of 1. These nodes randomly select a time-frequency resource block to reply SRES frames from multiple subchannels and multiple sub-slots. As shown in Figure 8, the subchannel1-1, subchannel1-$N_{R}$ and subchannel2-2 time-frequency resource blocks are selected to reply SRES frames. In this case, there is no collision. The SRES frame contains the identification and the sector index of transmitting SREQ frame node. After successfully receiving the SRES frame, nodes will update its neighbor list. SRES frames contain $N_{SRES}$ mini-slots and $N_{R}$ resource blocks on the time axis. The number of subchannels can be set flexibly according to the requirement and complexity of equipment.
4. Step 4—acknowledging SACK frame:
  After receiving the SRES frame, the node should reply the SACK frame. SACK frame contain $N_{SACK}$ mini-slots. Then, the entire three-way handshake process is completed.

It should be noted that: firstly, in order to ensure the synchronization of sector rotation, as shown in Figure 8, the total number of mini-slots in each sector process $N_{All}$ is equal to $\left(CW-1\right)+N_{SREQ}+1+N_{SRES}\;\cdot \;N_{R}+1+N_{SACK}=CW+N_{SREQ}+N_{SRES}\;\cdot \;N_{R}+N_{SACK}+1$. Secondly, BD-SBA algorithm, as the neighbor discovery part, can be applied to all kinds of ad-hoc network transmission protocols based on time division multiple access (TDMA). If the network transmission protocol needs reservation and data transmission, SRES and SACK frame can carry communication reservation information. At last, due to backoff procedure in step 1 and the adoption of multi-subchannel and multi-slot technology in step 3, the transmission time of SRES frames will increase. But because the advances in broadband technology and physical layer modulation technology provides a powerful technical guarantee for multi-subchannel and multi-slot technology.

Algorithm 1 is the BD-SBA algorithm in pseudocode form. As Algorithm 1 shown, for any node, the basic unit of algorithm is mini-slot, and the total number of mini-slots in one sector is $N_{All}$, which is equal to the sum of the maximum backoff value and the mini-slots of each frame. In one slot of a scanning beam, a node selects the backoff counter value first and starts to listen to the physical carrier energy. When the node detects the physical energy in the beam, it sets its mode to receiving and waits for the SREQ frame transmitted from other nodes. Otherwise, if the node does not sense any physical energy until the end of the backoff, it sets its mode to transmitting, and transmits the SREQ frame and starts the process of three-way handshake. The processing methods of receiving SREQ, SRES and SACK frames are as line 19, 25 and 31 of Algorithm 1. When the algorithm ends, the antenna beam will rotate to the next sector, and repeat the above process.

**Algorithm 1** Bi-Directional Scan-Based Algorithm (BD-SBA) Algorithm.**Initialization:** Set mini-slot counter (MSC) = 0, BC = 0, mode = recv and initialize CW, $N_{SREQ}$, $N_{SRES}$, $N_{SACK}$, $N_{R}$ and $N_{All}$; /* The “recv” means the mode of receiving, and the “tran” means the mode of transmitting. MSC is the mini-slot counter. BC is the value of the backoff counter, which is randomly selected between 0 and CW-1. */1:**while**$MSC<N_{All}$**do**2: **while** BC>0 **do**3:  **if** Carrier-sensing medium is idle **then**4:   BC=BC−1;/* Backoff procedure.*/5:   MSC=MSC+1;6:  **else**7:   mode=recv;/*Set mode to receiving.*/8:   BC=0;9:   Break.10:  **end if**11: **end while**12: **if** BC==0 **then**/*Sense nothing during backoff.*/13:  mode = tran;14:  Transmit SREQ;15:  mode=recv;16:  $MSC=MSC+N_{SREQ}+1$;17: **else**/*Sense energy.*/18:  **if** Receiving frame successfully **then**19:   **if** Frame is SREQ **then**20:    mode = tran;21:    Transmit SRES;22:    mode = recv;23:    $MSC=MSC+N_{SRES}\;\cdot \;N_{R}+1$;24:   **end if**25:   **if** Frame is SRES **then**26:    mode = tran;27:    Transmit SACK;28:    mode = recv;29:    $MSC=MSC+N_{SACK}+1$;30:   **end if**31:   **if** Frame is SACK **then**32:    MSC=MSC+1;33:   **end if**34:  **end if**35: **end if**36:**end while**

### 2.5. The Implementation Issues of BD-SBA Algorithm

In Section 2.3 and Section 2.4, we introduce the protocol of our BD-SBA algorithm. Compared with traditional algorithms, our algorithm is more complex. The complexity is mainly reflected in the equipment of bi-directional antennas and transceivers, and the overhead of the mini-slots in the scan. It is summarized as follows:

1. Compared with the traditional neighbor discovery algorithm, our BD-SBA algorithm adopts bi-directional antennas with synchronous rotation, which increases the complexity of the antenna feeder subsystem of the node.
2. Our algorithm requires two sets of transceivers to simultaneously listen on the whole channel and transmit and receive data on multiple sub-channels.
3. Due to the adoption of backoff and multi-slot mechanism in BD-SBA algorithm, the number of the mini-slots is more than that of traditional algorithm. The difference between them is $N_{SRES}\;\cdot \;\left(N_{R}-1\right)+CW-1$.

However, nowadays the advances in microwave devices and high speed digital processing technology can support the application of our algorithm:

Firstly, the new bi-directional antenna technology is developing rapidly. Bi-directional high gain dipole array antenna with good shaped patterns for WLAN applications has been designed and implemented in [17,18]. Nowadays, the gourth generation (4G) wireless communications systems has been commercialized. Multiple-input and multiple-output (MIMO) technology with multiple antennas has been accepted as one of key technologies in 4G systems [21]. Furthermore, massive MIMO technology with more antennas are the research hotspots of the fifth generation (5G) wireless communications systems in the future. Therefore, although our BD-SBA algorithm using bi-directional antenna technology increases the equipment complexity, the rapid development of antenna technology can support the industrial application of our proposed algorithm.

Secondly, orthogonal frequency division multiple access (OFDMA) using multiple sub-channels technology has been studied for many years. For OFDMA using in LTE cellular network, the whole channel can be divide into many sub-carriers, and they can be grouped into many resource blocks (RBs) which are allocated to user terminals (UEs). Furthermore, for the next-generation wireless local area networks (WLANs), Institute of Electrical and Electronics Engineers (IEEE) 802.11ax, OFDMA technology has been adopted in the draft [22]. Therefore, we believe that the technology of multi sub-channel transceiver are mature.

Thirdly, high-speed signal processing devices which use in wireless communications are quite common. Therefore, the extra mini-slots overhead will only increase the complexity of hardware implementation, but will not have a significant impact on algorithm performance.

## 3. Algorithm Analysis

### 3.1. Theoretical Model

Assuming that the nodes in the scene are randomly distributed, *N* represents the number of neighbor nodes of every node. *N* is related to node distributed density, transmission power and channel model of nodes. That is, *N* represents the number of receiving nodes whose receiving power exceed the receiving threshold, that is, the number of nodes that can be “perceive” around the node. *M* represents the average number in one sector, and θ represents the degree of sector, and $N=M\;\cdot \;\frac{2\pi }{\theta }$. For each node, assuming that the discovery of each sector is independent, it means that the discovery probability of one sector is not different from that of another sector. We set D(t) as the number of discovered neighbor nodes in one sector and D(t)≤M. The *t* means the $t_{th}$ scan. Since a scan contains multiple sectors, we can study the performance of a single scan equivalently by studying the performance of a single sector, assuming that the discovery of each sector is independent.

For the SBA algorithm, $P_{SBA}\left(t\right)$ is defined as in the $t_{th}$ scan the probability which node *i* can discover its undiscovered neighbor node, node *j*, when D(t−1) nodes have been discovered in the previous ${\left(t-1\right)}_{th}$ scan. We have [13]

(1) $$P_{SBA}\left(t\right)=2\;\cdot \;P_{t}\;\cdot \;\left(1-P_{t}\right)\;\cdot \;{\left(1-P_{t}\right)}^{M-1}\;\cdot \;{P_{t}}^{M-D(t-1)-1}.$$

As shown in Equation (1), the transmission probability of the SBA algorithm is a fixed value ($P_{t}$), which has been determined before scanning, while the transmission probability of our algorithm is based on bi-directional carrier sensing, and the probability is related to the maximum of the backoff contention window CW and the average number in one sector of a node (*M*). We define $P_{bk}$ as the transmission probability based on bi-directional carrier sensing, we have

(2) $$P_{bk}=\sum_{i=1}^{CW}\frac{1}{CW}\;\cdot \;(\frac{CW-i}{CW}{)}^{2M}.$$

$P_{bk}$ can be equivalent to the probability that the central node and the 2M nodes in the relative two sectors of the central node simultaneously select a random number in the range [0, CW-1], while the number selected by central node is the smallest. For example, if the backoff value of the center node is zero (possibility is $\frac{1}{CW}$), according to Equation (2), when the backoff values of the other nodes are larger than 0, the transmission possibility of the center node is ${\left(\frac{CW-1}{CW}\right)}^{2M}$ (i=1). If the backoff value of the center node is 1 (possibility is $\frac{1}{CW}$), when the backoff values of the other nodes are larger than one, the transmission possibility of the center node is ${\left(\frac{CW-2}{CW}\right)}^{2M}$ (i=2). Similarly, when *i* traverses other values, $P_{bk}$ is the sum of all of them. In particular, when *M* equals 0, there is no competition between the central node and the nodes in the relative two sectors of the central node, and the probability $P_{bk}$ is 1, which means that the central node must be in the transmitting mode.

In our BD-SBA algorithm, in the $t_{th}$ scan, node *i* can found node *j* if the followings are true:

1. The probability that node *i* is in transmitting mode is $P_{bk}$;
2. The probability that node *j* is in receiving mode is $1-P_{bk}$;
3. M−1 nodes in the sector of receiving node *j* are all in the receiving mode except node *i*, otherwise the collision of transmitting SREQ frames will occur, the probability is ${\left(1-P_{bk}\right)}^{M-1}$;
4. Node *j* randomly selects a time frequency resource block to transmit SRES frame, and the probability not conflict with other nodes is ${\left(1-\frac{1}{N_{tf}}\right)}^{M-D(t-1)-1}$, where $N_{tf}$ is the number of time frequency resource blocks, and these “other nodes” do not include node *i* and the D(t−1) found nodes already.

For the BD-SBA algorithm, $P_{BD-SBA}\left(t\right)$ is defined as in the $t_{th}$ scan the probability which node *i* can find its undiscovered node *j*, when D(t−1) nodes have been discovered in the previous scan (t−1). We have

(3) $$\begin{array}{c} P_{BD-SBA}\left(t\right)=2\;\cdot \;P_{bk}\;\cdot \;\left(1-P_{bk}\right)\;\cdot \;{\left(1-P_{bk}\right)}^{M-1}\;\cdot \;\\ (1-\frac{1}{N_{tf}}{)}^{M-D(t-1)-1}. \end{array}$$

**Figure 9:**
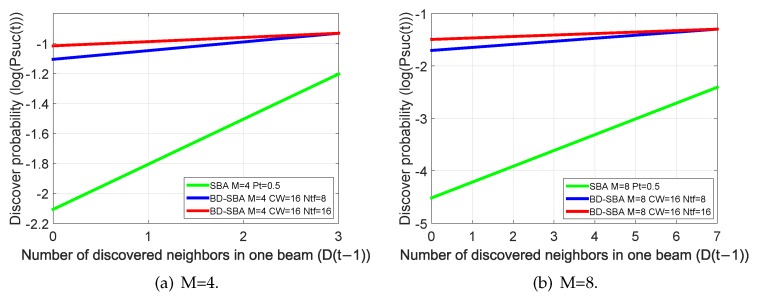
Comparison of discovery probability between SBA and BD-SBA algorithms.

In order to compare the difference between the SBA algorithm and the proposed BD-SBA algorithm in discovery probability, the numerical curves of Equations (1) and (3) under different conditions are shown in Figure 9. Figure 9a shows the values of two algorithms with CW=16, $N_{tf}=8$ and 16, M=4, and Figure 9b shows the values with CW=16, $N_{tf}=8$ and 16, M=8. As shown in Figure 9, firstly, the discovery probability of our BD-SBA algorithm in two cases of M=4 and 8 is far greater than that of SBA algorithm. Secondly, when M is changed from four to eight, the discovery probability of SBA decreases rapidly, but our BD-SBA algorithm decreases little. Again, $N_{tf}$ changed from eight to 16, and the discovery probability of BD-SBA algorithm increased slightly.

From Figure 9 and Equations (1) and (3), the BD-SBA algorithm’s discovery probability $P_{BD-SBA}\left(t\right)$ is greater than that of SBA, for three reasons:

1. The number of nodes *M* in one sector is very sensitive to the SBA algorithm, the main reason is that $P_{t}$ is not a small value (such as setting $P_{t}$ to 0.5), then the impact of M-power operation of ${\left(1-P_{t}\right)}^{M-1}$ is great; But the $P_{bk}$ in ${\left(1-P_{bk}\right)}^{M-1}$ is a small value when M≥3 (for example, when CW is 16, *M* is 4, $P_{bk}$ is 0.0825), so the M-power operation has little effect. While ${\left(1-P_{t}\right)}^{M-1}$ and ${\left(1-P_{bk}\right)}^{M-1}$ of two algorithms are the key factors that affect the collision of SREQ frames;
2. The discovery probability of BD-SBA algorithm does not change much when $N_{tf}$ change from 8 to 16, so long as the number of time-frequency resource blocks is not too small. That means our BD-SBA algorithm has a high probability of receiving SRES frame (from Equation (3) when M=4, D(t−1)=0, $N_{tf}=16$, the probability ${\left(1-\frac{1}{N_{tf}}\right)}^{M-D(t-1)-1}$ is 0.824), but if there are more than two nodes in one sector in the SBA algorithm, the collision of receiving SRES frames occur;
3. From the theoretical point of view, the ${\left(1-P_{bk}\right)}^{M-1}$ and ${\left(1-\frac{1}{N_{tf}}\right)}^{M-D(t-1)-1}$ in Equation (3) are exactly the key to overcome the two kinds of “link collision” problems of SBA algorithm.

### 3.2. Validation of Theoretical Model

In order to verify the theoretical model, the discovery ratio of the neighbor nodes in $t_{th}$ scan is used to evaluate the algorithm. We define

(4) $$E\left[N\left(t\right)\right]=\frac{\frac{2\pi }{\theta }\;\cdot \;\sum_{m=1}^{\min(M,t)}mP(m,t)}{N-1},$$

where E[N(t)] means the variation trend of discovery ratio with the increase of the scan t. The meaning of P(m,t) is that in one sector, the probability of discovering m nodes in the $t_{th}$ scan. The number of neighbor nodes in a sector is *M*.

A node can discover m neighbor nodes within t scans in a sector in the following two ways [5]:

1. Node *i* discovers *m* nodes in previous t−1 scans, and in $t_{th}$ scan, none of the remaining M−m nodes is discovered.
2. Node *i* discovers m−1 node in previous t−1 scans and any another node is discovered in the remaining M−m−1 node in the $t_{th}$ scan.

Therefore, P(m,t) can be expressed as: [5]

(5) $$P\left(m,t\right)=k_{1}\;\cdot \;P\left(m,t-1\right)+k_{2}\;\cdot \;P\left(m-1,t-1\right),$$

(6) $$k_{1}=1-\left(M-m\right)\;\cdot \;Psuc\left(t|D\left(t-1\right)=m\right),$$

(7) $$k_{2}=\left(M-m+1\right)\;\cdot \;Psuc\left(t|D\left(t-1\right)=m-1\right).$$

According to Equations (1), (3), (5)–(7), the E[N(t)] value of the SBA algorithm and the BD-SBA algorithm in this paper can be obtained by numerical calculation. In order to verify the theoretical model, the simulation scenario is as follows: The node number of the simulation scenario is 320, the sector angle is 45 degrees, there are eight sectors in one node, and the maximum perception range of nodes is r=100 m. The simulation scene is a circle with a radius of $R=100\sqrt{10}$. The node density is $320/\pi R^{2}$, and the average number of neighbor nodes is $320/\pi R^{2}\times \pi r^{2}$. Because there are 8 sectors of one node, the number of nodes in each sector is M=4. We used four sub-channels, four sub-slots, then $N_{tf}=16$, and we set CW as 16.

**Figure 10:**
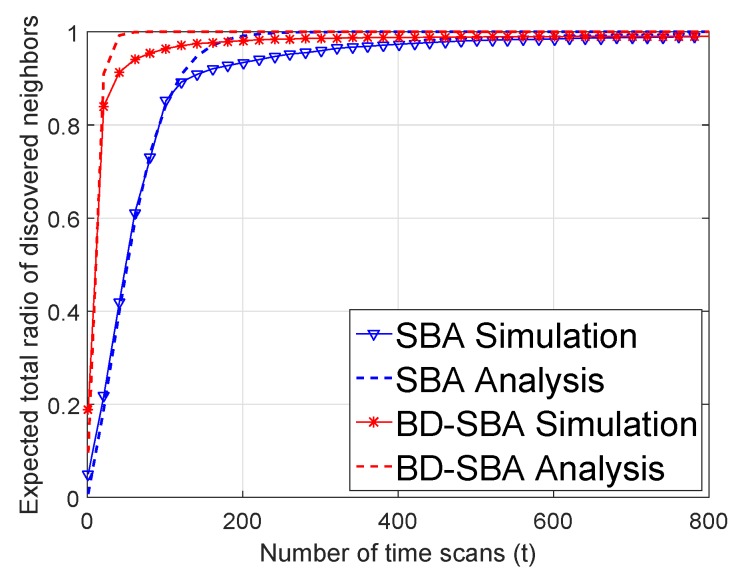
Validation of analysis.

The theoretical and simulation results are shown in Figure 10. As shown in Figure 10, the theoretical model and simulation results match well.

## 4. Simulation Results and Discussions

From the design of the protocol in Section 2 and the theoretical analysis in Section 3, we can see that the BD-SBA algorithm using bi-directional carrier sense collision avoidance and multi-subchannel has better anti-link collision performance.

First, we changed the sector degree to verify the performance of our BD-SBA algorithm. The simulation scene was 600 m × 600 m, and the 360 nodes were randomly distributed in the cell. The perception range of nodes was 100 m. The transmission probability $P_{t}$ of the SBA algorithm was set to 0.5. We set the sector degree of the two algorithms to 30, 45, 60 and 90 degrees, and observed the discovery ratio curves of the two algorithms.

**Figure 11:**
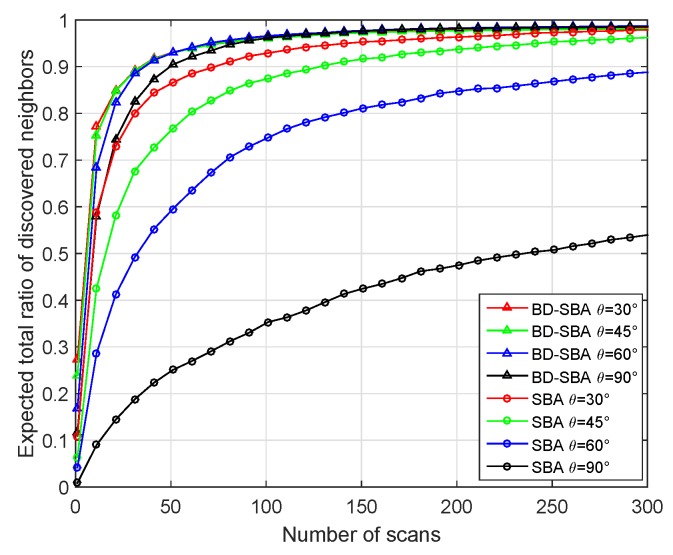
Performance of different sector degree.

From Figure 11, we can see that the performance of the SBA algorithm becomes worse as the sector degree increases. For SBA algorithm, the increase of sector degree means the increase of the number of nodes in one sector. Since the transmitting probability of the SBA algorithm node is fixed at 0.5, the more transmitting nodes in the receiving node’s sector, the more unlikely the receiving node will receive the SREQ frame in the first handshake step. Similar to the first handshake step, in the second handshake step, many nodes in the sector of transmitting node need to reply SRES frames with a probability of 1, so these SRES frames will cause collisions. Therefore, the degree of the sector has great influence on the SBA algorithm. With the increase of sector degree, the performance of BD-SBA algorithm is not very different. The smaller the sector, the better the performance. Firstly, with the increase of sector degree, the transmission probability $P_{bk}$ of BD-SBA decreases sharply due to the mechanism of bi-directional carrier sense collision avoidance, so that the collision of receiving SREQ frames will not increase. Second, even if the sector degree increases, because the SRES frames are transmitted on the time-frequency resource blocks, the transmitting node can find more than one node at the same time, so when the sector degree increases, the link collision of second handshake is small. In a word, the increase of sector degree will neither increase the collision of receiving SREQ frames of the first handshake step nor increase the collision of SRES frame of the second handshake step. Therefore, our BD-SBA algorithm is insensitive to the change of sector degree and has excellent performance.

Table 1 gives a comparison of the scanning times of the two algorithms in different sector degree. It can be seen from the table that whether most of the neighbor nodes (80%) or almost all of the neighbor nodes (98%) are found, the scanning times of the BD-SBA algorithm in this paper are less than the SBA algorithm, especially when the sector degree is large, the performance gain is obvious.

**Figure 12:**
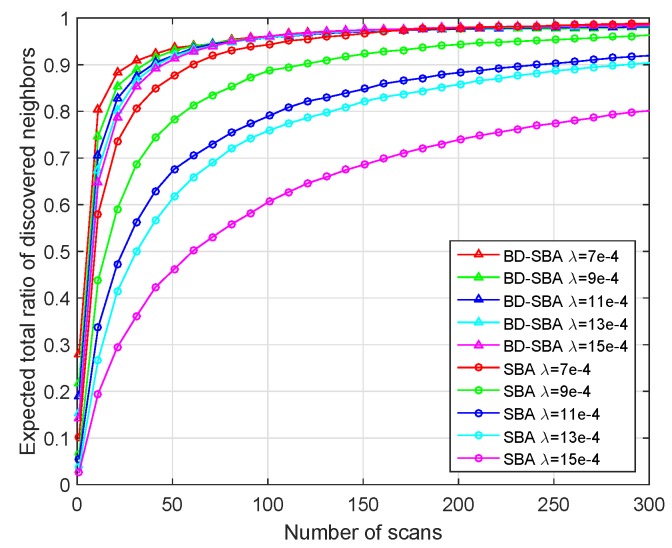
Performance of different node density.

Secondly, we change the number of nodes in the scene (equivalent to changing the node density), set different node densities $7\times 10^{-4}$, $9\times 10^{-4}$, $11\times 10^{-4}$, $13\times 10^{-4}$ and $15\times 10^{-4}$, and observe the discovery ratio curves of the two algorithms in Figure 12.

As shown in Figure 12 and Table 2, for SBA algorithm, with the increase of node density, the scanning time required by discover nodes becomes longer and longer, and the performance becomes worse. For the SBA algorithm, the increase of node density has the same effect as the increase of sector degree. As the number of nodes in a sector increases, it is more difficult to receive SREQ frames and SRES frames because of the two kinds of “link collision” problem. Therefore, the performance of the SBA algorithm in the dense scenario is poor. The BD-SBA algorithm can effectively overcome the impact of two kinds of “link collision” problems in dense scenarios by using the mechanism of bi-directional carrier sense collision avoidance and multi-subchannel. The algorithm is insensitive to node density and has excellent performance.

Thirdly, we simulated the impact of the number of time-frequency resource blocks on the algorithm. We set the sector degree to 45 degree, and the number of nodes to 360, and change the number of time-frequency resource blocks.

**Figure 13:**
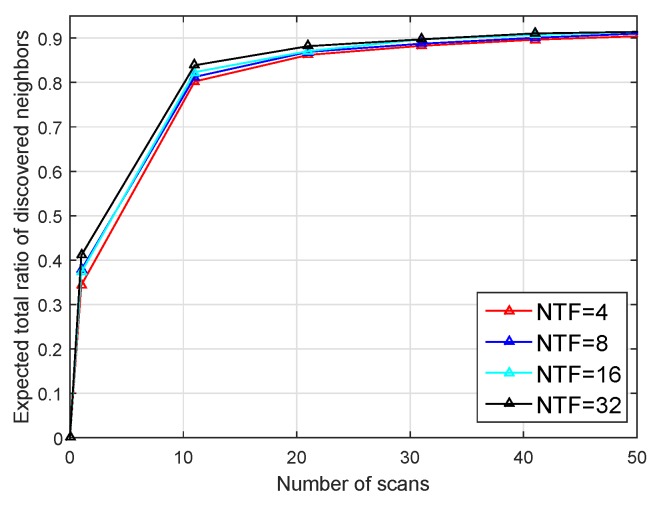
Performance of different number of time-frequency resource blocks.

As shown in Figure 13, different time-frequency resource blocks have little effect on the performance of the algorithm. The performance of more time-frequency resource blocks is slightly improved. The reason is that when the number of nodes in each sector is small (M<=4), even if the number of time-frequency resource blocks is small (e.g., four), the collision caused by receiving SRES frames will not be too large. The simulation results can be used to guide the future practical application.

**Figure 14:**
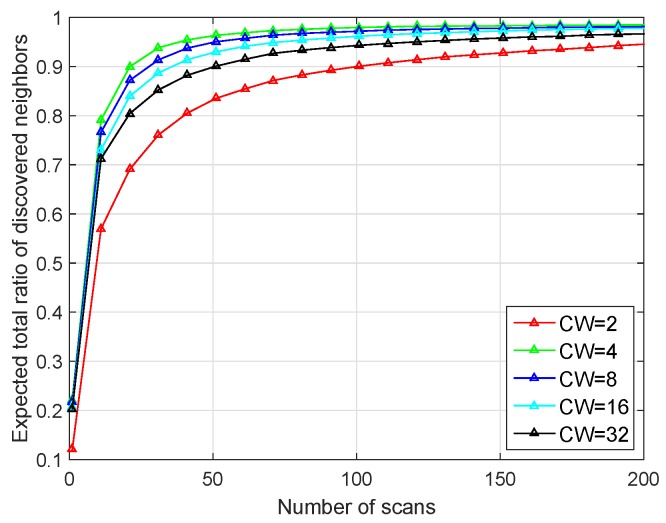
Performance of different values of the backoff window.

Fourthly, we simulated the impact of the value of backoff window CW on our BD-SBA algorithm. We set the value of backoff window to 2/4/8/16/32, and the other simulation parameters were the same as those in Figure 14.

As shown in Figure 14, when CW is four, performance is the best, and when CW is 8/16/32, the performance decreases, the performance of CW = 2 is the worst. According to Equation (2), when CW is 2/4/8/16/32, the transmitting probability $P_{bk}$ are 0.002/0.026/0.058/0.082/0.096 respectively. From Equation (3) it can be seen that with the increase of CW, the increase of $P_{bk}$ can exacerbate the first kind of “link collision” problem when the node receives SREQ frame, but when the CW is too small (CW = 2), the transmission probability of the node will be too low, thus affecting the performance of the algorithm.

In mobile wireless ad hoc sensor networks (MWASNs), the nodes in the networks may keep moving for some reason. Therefore, we verify the effect of the mobility model on our proposed algorithm by simulation. The simulation scene was 600 m × 600 m, and the 360 nodes were randomly distributed in the cell. The perception range of the node was 100 m. The above scenario was similar to small enemy environmental reconnaissance. We set the sector degree to $45^{\circ }$ and the physical layer data transmission rate to 60 kbps. The SREQ, SRES and SACK frames contained three bytes and occupied four mini-slots, and the transmission time of one mini-slot is 100 μs. The number of mini-slots of CW and $N_{R}$ was 16 and four, respectively. Therefore, according to the detailed procedure of our BD-SBA algorithm in Section 2.4, one scan includes 164 mini-slots. We assume that the nodes adopt Random Walk mobility model. For the random walk mobility model, each node randomly chooses a direction and move at a constant speed *V*, and $V\in [0,V_{\max}]$ [23]. We set $V_{\max}$ to 0 m/s (no movement), 20 m/s, 40 m/s, 60 m/s, 80 m/s respectively, and observe the performance of our algorithm taking care of the mobility of nodes.

**Figure 15:**
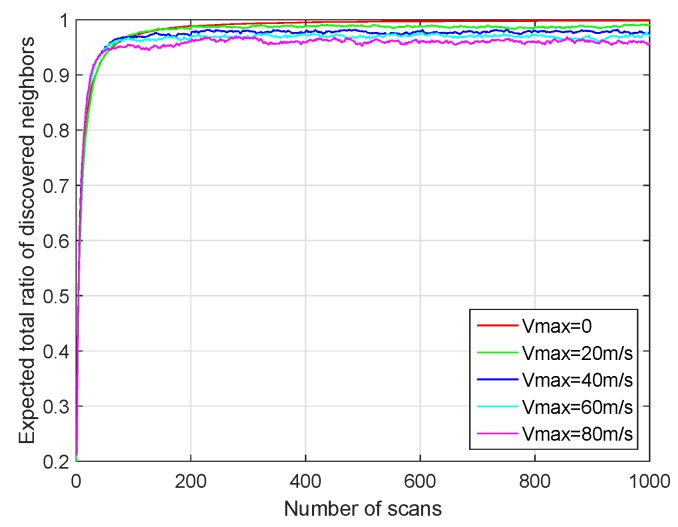
Performance with different moving speed.

As shown in Figure 15, the total ratio of discovered nodes increases with the number of scans. For different speeds, our algorithm can ensure that almost all the neighbor nodes can be discovered in a relatively short time (less than 200 scans). When the node does not move ($V_{\max}=0$), the total ratio of discovered nodes monotonically increases. However, with the increase of moving speed, the ratio decreases and the fluctuation of the ratio curve increases. The reason is that when the mobility of the nodes increases, the nodes continuously move out or into the perception range of their neighbor nodes, and then the stored neighbor nodes lists keep changing. This will lead to the loss and rediscovery of the nodes. However, as shown in Figure 15, although the mobility of nodes has a certain impact on the discovery ratio, the efficiency of the algorithm is relatively high.

**Figure 16:**
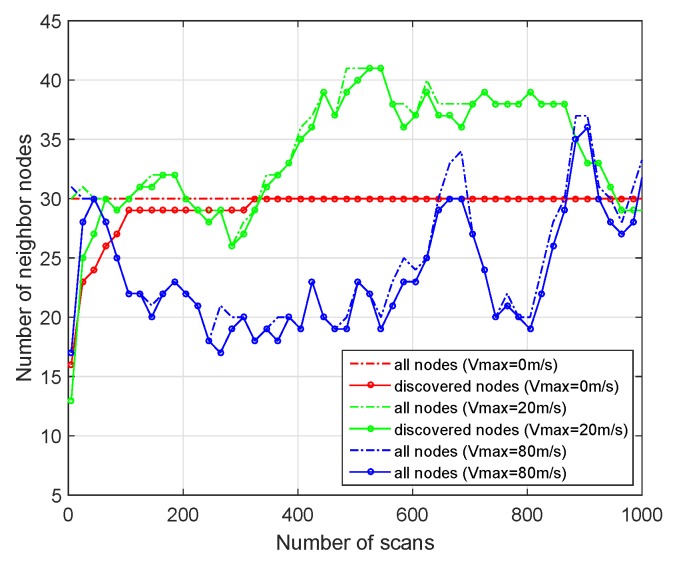
The number of discovered nodes for one single node with different moving speed.

In Figure 16, we select a single node with different speed in the simulation scene as an example to observe the discovered number of neighbor nodes. The solid line in the figure is the number of all nodes in the perception range of the single node, and the triangle sign is the number of discovered nodes. As shown in Figure 16, when all nodes don not move, the number of all nodes in the perception range of the selected node is a fixed value, and the number of discovered nodes increases with the increase of scans. When the moving speed increases, the number of all nodes in the perception range of the selected node changes constantly, and the greater the speed, the greater the fluctuation of the change. However, as shown in Figure 16, although the number of discovered nodes is always smaller than that of all neighbor nodes, the number of discovered nodes can always keep up with the changes of the number of all nodes. Therefore, our algorithm can support the mobility of nodes very well.

The mobility of nodes is mainly reflected in that the mobile nodes will continue to travel through the perception range of the surrounding nodes. The impact of mobility on the neighbor discovery algorithm is closely related to the discovery time. The smaller the time, the better the algorithm. Therefore, we can use the time of discovering neighbor nodes to estimate roughly the moving speed supported by an algorithm.

Take our simulation for example. As shown in Figure 15, if we set the mini-slot time supported by hardware to 100 μs, the time (about 200 scans, one scan includes 164 mini-slots) of discovering almost all of the neighbor nodes is 3.28 s. The perception range of the node is 200 m (the perception radius is 100 m). When the moving speed of the node is less than $\frac{200\;m}{3.28\;s}\approx 61$ m/s, the node can just pass through the perception range of its neighbor node and can not be lost. Therefore, we believe that our algorithm can be applied when the node speed is not greater than 61 m/s in our simulation scenario. The analysis of our proposed algorithm taking care of the mobility of nodes in other scenarios is similar. Moreover, with the development of digital signal processor, mini-slot time can be smaller, so our algorithm has better support for the mobility of nodes.

## 5. Conclusions

This paper first summarizes two kinds of “link collision” problems that restrict the performance of classical SBA algorithm. Aiming at these two “link collision” problems, a neighbor discover BD-SBA algorithm based on bi-directional carrier sense collision avoidance and multi-subchannel multi-slot is proposed. The protocol design, theoretical model and simulation verification are given. The theoretical model and simulation results show that the BD-SBA algorithm has excellent neighbor discovery performance and anti-link collision advantages. In summary, it is believed that the proposed BD-SBA algorithm will pave a new technical way to investigate problems of neighbor discovery in wireless ad-hoc sensor networks. In particular, the author’s laboratory has carried out research on the application of multi-channel in DTRA protocol for reservation and data transmission stage [24,25]. In the future, our neighbor discovery algorithm combined with previous research [24,25] can significantly improve the performance of the DTRA protocol at all stages.

## Figures and Tables

**Table 1:** Scan times of different sector degrees.

Discover Radio	Sector Degree	SBA	BD-SBA
80% nodes	$30^{\circ }$	30	13
	$45^{\circ }$	60	15
	$60^{\circ }$	145	21
	$90^{\circ }$	1570	28
98% nodes	$30^{\circ }$	250	230
	$45^{\circ }$	700	233
	$60^{\circ }$	1840	235
	$90^{\circ }$	5230	240

**Table 2:** Scans times of different node densities.

Discover Radio	Node Density	SBA	BD-SBA
80% nodes	$7\times 10^{-4}$	30	13
	$9\times 10^{-4}$	55	15
	$11\times 10^{-4}$	105	18
	$13\times 10^{-4}$	130	20
	$15\times 10^{-4}$	300	25
98% nodes	$7\times 10^{-4}$	423	265
	$9\times 10^{-4}$	650	268
	$11\times 10^{-4}$	1245	270
	$13\times 10^{-4}$	2300	272
	$15\times 10^{-4}$	4650	275
